# Iron Deficiency Anemia With a Life-Threatening Low Hemoglobin Level

**DOI:** 10.7759/cureus.20150

**Published:** 2021-12-04

**Authors:** Abdulrahman M Alateeq, Hessa A Alshammari, Abdulmalik M Alsaif

**Affiliations:** 1 Family Medicine, King Saud Bin Abdulaziz University for Health Sciences College of Medicine, Riyadh, SAU; 2 Internal Medicine, King Khalid Hospital, Hail, SAU

**Keywords:** menorrhagia, life-threatening hemoglobin level, severe anemia, uterine fibroids, iron deficiency anemia

## Abstract

Iron deficiency anemia (IDA) accounts for roughly half of all anemia cases globally. Menorrhagia and menstrual cycle disorders render women of childbearing age more prone to IDA. One of the leading causes of menorrhagia is uterine fibroids (leiomyomas), which are defined as hyperplastic, usually benign, overgrowths of uterine wall muscle and connective tissue. This is a case report of a 45-year-old woman who came to the emergency department complaining of progressive fatigue and dizziness for two months. She was admitted as a case of life-threatening IDA with a hemoglobin level of 3.0 g/dL. She was ambulatory and hemodynamically stable without any features of severe anemia. Pelvic ultrasound was performed and revealed uterine fibroids. After partial correction of the anemia, she underwent a total hysterectomy as a definitive treatment.

## Introduction

Iron deficiency anemia (IDA) is characterized by iron reservoir depletion and accounts for roughly half of all anemia cases globally [1]; it affects women twice as much as men, especially during the reproductive years [2]. Since most iron is stored as ferritin and circulates in the blood, blood loss is the most prevalent cause of IDA [3]. As such, premenopausal women are more prone to IDA than men and postmenopausal women. Studies suggest that premenopausal women’s iron storage would be approximately three times lower than women 10 years post-menopause [4]. The symptoms of IDA might vary depending on etiology and severity; nevertheless, the most typically reported symptoms are fatigue, pallor, weakness, and headache [5].

Menorrhagia and IDA are co-occurring pathologies in women of childbearing age. Menstrual problems that cause menorrhagia are thought to account for 5%-10% of females who present with IDA [6]. One of the leading causes of menorrhagia is uterine fibroids (leiomyomas), which are defined as hyperplastic, usually benign, overgrowths of uterine wall muscle and connective tissue [7,8]. It is prevalent in women of childbearing age but can also occur after menopause. Intramural fibroids are the most common kind and can appear as single or multiple tumors [9]. Patients usually present with irregular heavy periods and subsequently may develop IDA. Surgical removal with myomectomy or hysterectomy is the conventional therapy for symptomatic patients, depending on their intention to maintain fertility [10].

In this report, we present a rare case of a female patient with a severely low hemoglobin level, secondary to uterine fibroids. 

## Case presentation

We discuss a 45-year-old Saudi woman with no known chronic conditions. She came to the emergency department at King Khalid Hospital in Hail, Saudi Arabia (SA), complaining of progressive fatigue and dizziness for two months. Her fatigue was exacerbated by minimal physical activity and associated with palpitations and dyspnea. She also had a history of appetite decline and weight loss for six months. She denied any recent traumas, bleeding, fever, or night sweats. She denied any history of abdominal or pelvic pain. Apart from that, her past medical and surgical history was unremarkable, and she had no previous admissions or similar complaints. She was not on any medications. There was no history of similar conditions in the family. Regarding the patient's gynecological history, she stated that her menstrual periods had changed in the last two years. They became abnormally long, lasting for more than a week most of the time, with heavy fresh blood flow and intense menstrual pain. She had to change her pads more frequently than usual, but the frequency of her cycles was regular. Regarding her obstetric history, the patient had four normal pregnancies, with full-term deliveries by cesarean section, without complications. 

On clinical examination, she was conscious, alert, and oriented to time, place, and person. She showed no signs of dyspnea and was able to speak comfortably in full sentences. The patient had notably pale skin and conjunctivas. Her extremities were cold, but no nail changes were detected. Her vitals: temperature 36.9°C, blood pressure 96/50 mmHg, a pulse of 98 beats per minute, oxygen saturation (SpO2) 99 percent with room air, and a respiratory rate of 21 breaths per minute, with a weight of 50.7 kg, and a height of 155 cm. The abdominal examination revealed a soft and lax abdomen with no organomegaly or palpable masses. Both the cardiovascular and neurological examinations yielded normal findings. For the musculoskeletal and neurological examinations, the patient was ambulatory, with bilateral, active, full-range motion in both upper and lower limbs, along with normal sensation. The patient was offered a pelvic examination, but she refused.

For initial assessment, a complete blood count (CBC) was done and revealed a severely low hemoglobin level of 3.0 g/dL. The result was verified after CBC repetition. A pregnancy test was ordered, which was negative. The patient was admitted under medical care. Table 1 summarizes CBC laboratory results ordered upon her arrival. Hematocrit was 12.0%, and hemoglobin was severely low at 3.0 g/dL. Her results for mean corpuscular volume (MCV), mean corpuscular hemoglobin (MCH), and mean corpuscular hemoglobin concentration (MCHC) were 59.1 fL, 14.8 pg, and 25.0 g/dL, respectively. The red cell count was 2.03 x10^12^/L, with a red cell distribution width (RDW) of 19.5%. These findings, with the peripheral blood smear, were consistent with microcytic hypochromic anemia.

**Table 1:** Complete blood count done upon patient’s arrival MCV: mean corpuscular volume; MCH: mean corpuscular hemoglobin; MCHC: mean corpuscular hemoglobin concentration; RDW: red cell distribution width

Laboratory	Result	Reference range
Hemoglobin	3.0 g/dL	12 - 15.5 g/dL
Hematocrit	12 %	34.9 - 44.5 %
Red cell count	2.03 x10^12^/L	3.9 - 5.03 x10^12^/L
MCV	59.1 fL	81.6 - 98.3 fL
MCH	14.8 pg	26.5 - 32.6 pg
MCHC	25.0 g/dL	32 - 36 g/dL
RDW	19.5 %	11.9 - 15.5 %
Total leukocytic count	4.25 x10^9^/L	3.5 - 10.5 x10^9^/L
Basophils absolute count	0.06 x10^9^/L	0 - 0.3 x10^9^/L
Eosinophils absolute count	0.06 x10^9^/L	0.05 - 0.5 x10^9^/L
Neutrophils absolute count	2.04 x10^9^/L	1.7 - 7 x10^9^/L
Lymphocytes absolute count	1.69 x10^9^/L	0.9 - 3.1 x10^9^/L
Platelet count	574 x10^9^/L	150 - 450 x10^9^/L

Table 2 shows chemistry lab results. The serum ferritin level was extremely low, at 1.93 ng/mL. Further investigations, including coagulation profile, liver function test (LFT), fecal occult blood test, and chest X-ray, were all unremarkable.

**Table 2:** Chemistry done upon patient’s arrival

Laboratory	Results	Reference range
Blood urea nitrogen	9.0 mg/dl	6 - 20 mg/dl
Creatinine in serum	0.63 mg/dl	0.51- 0.95 mg/dl
Uric acid in serum	2.0 mg/dL	2.4 - 5.7 mg/dL
Chloride in serum	111 mmol/L	98 - 107 mmol/L
Potassium (K) in serum	4.4 mmol/L	3.5 - 5.1 mmol/L
Sodium (Na) in serum	140.0 mmol/L	136 – 145 mmol/L
Calcium in serum (Total)	8.8 mg/dl	8.6 - 10.2 mg/dl
Globulin in serum	2.80 g/dl	2 - 3.9 g/dl
Estimated glomerular filtration rate (eGFR)	>60 ml/min/1.73 m^2^	>60 ml/min/1.73 m^2^
Glucose in plasma (Fasting)	121 mg/dl	70 - 100 mg/dl
Bilirubin (Total)	1.20 mg/dL	0.1 - 1.2 mg/dL
Bilirubin (Direct)	0.50 mg/dl	0.02 - 0.3 mg/dl
Alanine aminotransferase (ALT)	11 U/L	5 - 31 U/L
Aspartate aminotransferase (AST)	14 U/L	0 – 32 U/L
Alkaline phosphatase	69 U/L	35 – 104 U/L
Albumin in serum	4.2 g/dL	3.5 - 5.2 g/dL
Total protein in serum	7.0 g/dL	6.4 - 8.3 g/dl
T4 (Free)	0.98 ng/dL	0.7 - 1.48 ng/dL
Thyroid stimulating hormone (TSH)	0.64 uIU/mL	0.35 - 4.94 uIU/mL
CRP high-sensitive	0.07 mg/dl	<0.5 mg/dl
Cholesterol	90 mg/dl	(Optimal) <200 mg/dl
Triglycerides (TG) in serum	37 mg/dL	(Optimal) <200 mg/dL
High density Lipoprotein (HDL)	35 mg/dL	(Optimal) >60 mg/dL
Low density lipoprotein (LDL)	54 mg/dl	(Optimal) <100 mg/dl
Ferritin in serum	1.93 ng/mL	13 - 150 ng/mL
Iron (Fe) in serum	13.3 ug/dl	33 - 193 ug/dl
Vitamin D	11.2 ng/mL	20 - 40 ng/mL

**Figure 1:**
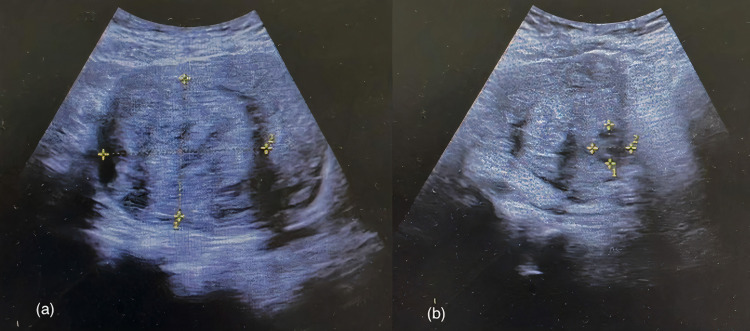
Transvaginal ultrasound showing intramural uterine fibroids (yellow markers) with sizes: (a) 5.63 x 6.70 cm (b) 1.68 x 1.68 cm

We performed a transvaginal ultrasound (Figure 1), revealing heterogeneous well-defined uterine lesions. The patient was eventually diagnosed with intramural uterine fibroids, complicated with life-threatening IDA.

During her two-day hospital stay, she was given four units of packed red blood cells. She received a total of 1,000 mg ferrous sulfate intravenous therapy, beginning during her hospital stay and continuing in the clinic after discharge. Before that, her hemoglobin level reached 9.1 g/dL. When she returned for a follow-up visit after one week, she felt much better, and her symptoms had resolved. She was referred to the gynecological department for definitive management, given oral iron supplements, multivitamins, and long-term follow-up. After partial correction of the anemia, the patient was admitted under gynecological care, with a total hysterectomy done.

## Discussion

To our knowledge, this is the lowest hemoglobin level in a hemodynamically stable patient in SA and one of few cases worldwide. The peculiarity of this case stems from the patient's hemodynamic stability despite a life-threatening hemoglobin level.

Although most cases of uterine fibroids are asymptomatic and do not require treatment, our patient experienced significant symptoms, causing her hemoglobin to drop to dangerously low levels [11]. The patient had been suffering for some time with menorrhagia and symptoms of anemia but did not seek medical help. This may be partly due to the absence of local symptoms with uterine fibroids, e.g., abdominal or pelvic discomfort or pain, usually reported in symptomatic patients. 

The hemodynamic stability of our patient might be explained by her gradual and extended history. A physiological compensating process might have occurred, preventing severe anemia consequences [12]. In 2013, a middle-aged woman with a history of menorrhagia had a hemoglobin level of 1.7 g/dL; however, she presented with altered mental status and was hemodynamically less stable than our patient [13]. A 45-year-old woman had an even lower hemoglobin level of 1.4 g/dL. Like our case, she was diagnosed with leiomyomas; however, she was unable to walk, with a history of a mechanical fall [14]. Another middle-aged woman with celiac disease complained of stomach discomfort and exhaustion [15]. Her hemoglobin level was 1.7 g/dL. She required mobility help, and her symptoms were more severe than our patient's. These uncommon examples of life-threatening hemoglobin levels and hemodynamic stability demonstrate how adaptive the physiologic compensatory systems can be with chronic anemias, yet they ultimately require medical care at some point.

## Conclusions

Critically low hemoglobin levels in hemodynamically stable patients have rarely been reported. This case is one of few reported worldwide. In the present case, a premenopausal female patient came to the emergency department with progressive fatigue and dizziness. Her initial blood tests revealed a life-threatening hemoglobin level, even though she did not complain of severe symptoms. Since they are usually co-occurring conditions, menstrual cycle disorders must be excluded in premenopausal women with symptoms of IDA.
